# Contrast Tempo of Movement and Its Effect on Power Output and Bar Velocity During Resistance Exercise

**DOI:** 10.3389/fphys.2020.629199

**Published:** 2021-01-20

**Authors:** Michal Wilk, Jakub Jarosz, Michal Krzysztofik, Aleksandra Filip-Stachnik, Marcin Bialas, Agata Rzeszutko-Belzowska, Adam Zajac, Petr Stastny

**Affiliations:** ^1^Institute of Sport Sciences, Jerzy Kukuczka Academy of Physical Education in Katowice, Katowice, Poland; ^2^Faculty of Physical Education, Gdańsk University of Physical Education and Sport, Gdańsk, Poland; ^3^College of Medical Sciences, Institute of Physical Culture Studies, University of Rzeszów, Rzeszów, Poland; ^4^Department of Sport Games, Faculty of Physical Education and Sport, Charles University, Prague, Czechia

**Keywords:** resistance exercise, cadence, time under tension, velocity of movement, duration of repetition

## Abstract

In this study, we examined the impact of contrast movement tempo (fast vs. slow) on power output and bar velocity during the bench press exercise. Ten healthy men (age = 26.9 ± 4.1 years; body mass = 90.5 ± 10.3 kg; bench press 1RM = 136.8 ± 27.7 kg) with significant experience in resistance training (9.4 ± 5.6 years) performed the bench press exercise under three conditions: with an explosive tempo of movement in each of three repetitions (E/E/E = explosive, explosive, explosive); with a slow tempo of movement in the first repetition and an explosive tempo in the next two repetitions (S/E/E = slow, explosive, explosive); and with a slow tempo of movement in the first two repetitions and an explosive tempo in the last repetition (S/S/E = slow, slow, explosive). The slow repetitions were performed with a 5/0/5/0 (eccentric/isometric/concentric/isometric) movement tempo, while the explosive repetitions were performed with an X/0/X/0 (X- maximal speed of movement) movement tempo. During each experimental session, the participants performed one set of three repetitions at 60%1RM. The two-way repeated measures ANOVA showed a statistically significant interaction effect for peak power output (PP; *p* = 0.03; *η*^2^ = 0.26) and for peak bar velocity (PV; *p* = 0.04; *η*^2^ = 0.24). Futhermore there was a statistically significant main effect of condition for PP (*p* = 0.04; *η*^2^ = 0.30) and PV (*p* = 0.02; *η*^2^ = 0.35). The *post hoc* analysis for interaction revealed that PP was significantly higher in the 2nd and 3rd repetition for E/E/E compared with the S/S/E (*p* < 0.01 for both) and significantly higher in the 2nd repetition for the S/E/E compared with S/S/E (*p* < 0.01). The *post hoc* analysis for interaction revealed that PV was significantly higher in the 2nd and 3rd repetition for E/E/E compared with the S/S/E (*p* < 0.01 for both), and significantly higher in the 2nd repetition for the S/E/E compared with the S/S/E (*p* < 0.01). The *post hoc* analysis for main effect of condition revealed that PP and PV was significantly higher for the E/E/E compared to the S/S/E (*p* = 0.04; *p* = 0.02; respectively). The main finding of this study was that different distribution of movement tempo during a set has a significant impact on power output and bar velocity in the bench press exercise at 60%1RM. However, the use of one slow repetition at the beginning of a set does not decrease the level of power output in the third repetition of that set.

## Introduction

Programming of resistance training involves the manipulation of numerous variables, including load, volume, exercises order or selection, and the rest intervals between sets (Schoenfeld et al., 2017a,b; Grgic et al., 2018; Nunes et al., 2020). However, the training variable that is often neglected but is essential to consider for achieving these goals is the movement tempo of particular repetitions. There are two types of movement tempo during resistance training: unintentional and intentional. The unintentional slow tempo can inadvertently occur during resistance training whereby a heavy load or the manifestation of fatigue is primarily responsible for a slower movement (i.e., increased duration of the repetition). Conversely, an intentional slow movement tempo can be purposefully used when the load is light enough to control it, and fatigue does not influence one’s ability to control the velocity of movement. Therefore, conscious control of the movement tempo is only possible to a certain extent (Suchomel et al., 2019a,b) during concentric actions, where strength is a limiting factor. The intentional movement tempo is often communicated using a sequence of digits (e.g., 4/0/2/0), where each digit defines the duration of a particular phase of the movement. According to the recommendations of Wilk et al. (2020d), a four-digit combination should be used, which describes the eccentric, isometric/transition, concentric, and isometric/transition phases (Wilk et al., 2020d). For example, 4/0/2/0 denotes a 4-s eccentric phase, no intentional isometric pause during the transition phase, a 2-s concentric phase, and no pause between the completion of the concentric phase and the beginning (eccentric phase) of the next repetition. The changes in movement tempo affect the number of performed repetitions in a single set (Sakamoto and Sinclair, 2006; Wilk et al., 2018a), the time under tension (TUT; Wilk et al., 2018a), and the maximum possible load lifted during a resistance exercise (Headley et al., 2011; Wilk et al., 2020a,b). As a result, previous research has considered different movement tempos, loads, and number of repetitions, showing that changes in these variables affect physiological, metabolic, and endocrine responses (Tanimoto and Ishii, 2006; Watanabe et al., 2013; Rogatzki et al., 2014; Wilk et al., 2018b). Further, the changes in movement tempo at a given external load can influence acute exercise volume, and in turn, the resultant changes in maximum strength, power, and hypertrophy (Keeler et al., 2001; Hunter et al., 2003; Hatfield et al., 2006; Sakamoto and Sinclair, 2006; Headley et al., 2011; Wilk et al., 2020d). According to the American College of Sports Medicine (2009), untrained individuals should use slow and moderate movement tempos. For intermediate sports level, it is recommended that moderate tempos should be used, and for advanced athletes, a variety of tempos, from slow to fast velocities are recommended. However, the combination of slow, moderate, and fast tempos for advanced training may provide the most benefits, depending on the load and the number of performed repetitions (Farthing and Chilibeck, 2003; Munn et al., 2005). Regarding hypertrophy, these guidelines generally concur with one fairly recent meta-analysis (Schoenfeld et al., 2015a), which indicates that similar hypertrophic responses occur when the repetition duration ranges from 0.5 to 8 s, which is a very wide range, whereby acute exercise stress could largely vary (Wilk et al., 2020d). The possible advantages of faster tempos on strength gains were mentioned in a meta-analysis by Davies et al. (2017), but the differences between strength gains following faster and slower tempos were not statistically significant.

Fast or explosive movement tempo is generally used to improve an individual’s power output (Baechle and Earle, 2008), which can be defined as the rate of performing work (force x velocity; McArdle et al., 2015). Despite the small number of studies analyzing the influence of different movement tempos on power output, it can be indicated that faster movement tempos are more effective at developing power (Morrissey et al., 1998; Fielding et al., 2002; Neils et al., 2005; Bottaro et al., 2007). However, the biomechanical demands required from the limbs change with the lifted load and to an extent depend on the respective joint (Kipp et al., 2011, 2013). The generation and translation of joint power are influenced by external load and athlete-dependent traits. This subsequently alters the power-load profile, explaining the broad spectrums of loads reported to optimize power output. Therefore, for power development, it is recommended to use fast or explosive movement tempo, yet using a combination of external loads based on assorted percentages of 1RM may be prescribed to optimize joint movement (Williams et al., 2018). The available data regarding the influence of different movement tempos on power output refers to chronic changes, and only one previous study considered the acute effects of movement tempo on power output and bar velocity (Wilk et al., 2019b). A study by Wilk et al. (2019a) showed that faster eccentric tempo (2/0/X/0 – X determines maximal speed) during the bench press exercise generates a higher level of power and greater bar velocity in the concentric movement compared to a slower eccentric tempo (6/0/X/0). Therefore, this study indicates that the duration of the eccentric phase of movement has a significant impact on power output and bar velocity during the concentric phase of the movement. However, a controlled movement tempo can be used only when the load is light enough to control the velocity of the bar and not when the load is light enough to allow fatigue to disturb the tempo of movement (Suchomel et al., 2019a,b). Therefore, increased fatigue during a set, especially when it is performed to muscular failure, may limit the ability to maintain the intended movement tempo. Furthermore, a constant movement tempo does not have to be used in every set or in every repetition. Variable movement tempo can be used during a particular set of a resistance exercise, in which the first repetitions (e.g., reps 1 and 2) are performed at a slower tempo and then the following ones (e.g., reps 3–6) at a faster tempo or vice versa. What is particularly important that all previous research has used a constant movement tempo throughout the sets or training session (e.g., 6/0/1/0) (Tanimoto and Ishii, 2006; Schoenfeld et al., 2015a; Davies et al., 2017), and currently, there are no studies which have assessed the effect of different distribution of movement tempo or contrast movement tempo (fast contrast to slow) within a set on the level of acute responses and chronic adaptive changes.

Given that power output is particularly significant for numerous sport disciplines, it would be interesting to examine whether a different distribution of movement tempo affects bar velocity and power output during resistance exercise. Since the widespread use of the bench press as a basic exercise for developing upper body strength and power output (Stastny et al., 2017; Wilk et al., 2019a, 2020c), the aim of the present study was to evaluate the effects of contrast movement tempo (fast vs. slow) on power output and bar velocity during the bench press exercise. It was hypothesized that contrast tempo significantly affects power output and bar velocity during the bench press exercise.

## Materials and Methods

The researchers examined the impact of different movement tempo distribution during a single set of the bench press exercise on power output and bar velocity. The experiment was performed following a randomized crossover design, where each subject performed three different testing protocols in random and counterbalanced order, 1 week apart: with an explosive tempo of movement in each of three repetitions (E/E/E = explosive, explosive, explosive); with a slow tempo of movement in the first repetition and an explosive tempo in the next two repetitions (S/E/E = slow, explosive, explosive); and with a slow tempo of movement in the first two repetitions and an explosive tempo in the last repetition of the set (S/S/E = slow, slow, explosive). The slow repetitions were performed with a 5/0/5/0 movement tempo, while the explosive repetitions were performed with an X/0/X/0 movement tempo. During each experimental session, the participants performed one set of three repetitions at 60%1RM. Before the main tests, one familiarization session was allowed. One week before the first main session, maximal bench press strength (1 repetition maximum-1RM) was evaluated. The following variables were measured using a linear position transducer: peak power output (PP), mean power output (MP), peak bar velocity (PV), and mean bar velocity (MV). All testing sessions were performed in the Strength and Power Laboratory at the Academy of Physical Education in Katowice, Poland.

### Participants

Ten healthy men (athletes representing, mixed martial arts *n* = 5; handball *n* = 3; athletics throws *n* = 2) with experience in resistance training (9.4 ± 5.6 years) volunteered for the study after completing an informed consent form (age = 26.9 ± 4.1 years; body mass = 90.5 ± 10.3 kg; bench press 1RM = 136.8 ± 27.7 kg). The main inclusion criteria were a personal best on the bench press of at least 120% body mass and that the subject was free from musculoskeletal injuries for at least 6 months before the study. The subjects were instructed to maintain their normal dietary habits over the course of the study for the duration of the experiment. They were informed about the benefits and potential risks of the study before providing their written informed consent for participation and were allowed to withdraw from the study at any time. The study protocol was approved by the Bioethics Committee for Scientific Research, the Academy of Physical Education in Katowice, Poland (10/2018), and all procedures were in accordance with the ethical standards of the Declaration of Helsinki, 1983.

### Procedures

#### Familiarization Session and the 1RM Strength Test

Two weeks before the main experiment, the participants performed a familiarization session. One week before the main experiment, the 1RM bench press test was performed. The participants arrived at the laboratory at the same time of day as the upcoming experimental sessions (in the morning between 9:00 and 10:00 am). On arrival, body mass was measured and then the participants performed a general upper body warm-up. For general upper body warm-up, the participants cycled on an ergometer for 5 min at an intensity that resulted in a heart rate of around 130 bpm, followed by a general warm-up of 10 body mass pull-ups and 15 body mass push-ups were performed. Next, the participants performed 15 and 10 bench press repetitions at a load of 20 and 40% of their estimated 1RM. After the warm-up, the familiarization session began. During the familiarization session, each subject performed three sets of three repetitions of the bench press at a load of 50% of their estimated 1RM. One set of each tempo was performed (E/E/E; S/E/E; S/S/E). The familiarization sessions were performed in order to minimize possible learning effects during the main tests.

During the 1RM test session, the general upper body warm-up was the same as during the familiarization session. Next, the participants performed 15, 10, and 5 bench press repetitions at a load of 20, 40, and 60% of their estimated 1RM. The first testing load was set to an estimated 80%1RM and was increased by 2.5–10 kg for each subsequent attempt. This process was repeated until failure. A licensed strength and conditioning coach supervised the 1RM test procedure and made decisions on load progression in subsequent attempts. During the 1RM test, the participants executed one repetition with volitional movement tempo (Wilk et al., 2020a,b). The rest interval between successful attempts was 5 min. Hand placement on the barbell was set at 150% of the individual bi-acromial distance, and this was used for all main attempts and all experimental sessions (Wilk et al., 2019a). No bench press suits, weightlifting belts, or other supportive garments were permitted. Three spotters were present during all attempts to ensure safety and technical proficiency.

#### Experimental Sessions

In a randomized and counterbalanced order, the subjects performed the bench press exercise under three testing conditions, which differed in tempo distribution during the set:

- E/E/E – 1st repetition explosive, 2nd repetition explosive, 3rd repetition explosive;
- S/E/E – 1st repetition slow, 2nd repetition explosive, 3rd repetition explosive;
- S/S/E – 1st repetition slow, 2nd repetition slow, 3rd repetition explosive.

During the explosive repetition, a X/0/X/0 movement tempo was used, while during the slow repetition protocol, the 5/0/5/0 movement tempo was used. During each testing protocol, the subject performed one set of the bench press at 60%1RM, following a metronome guided movement tempo (Korg MA-30, Korg, Melville, New York, USA). The external load and the number of performed repetitions was determined in accordance with the recommendation of American College of Sports Medicine (2009) concerning power training. A linear position transducer system (Tendo Power Analyzer, Tendo Sport Machines, Trencin, Slovakia) was used for the evaluation of bar velocity (Garnacho-Castaño et al., 2015). Measurements were made independently for each repetition and automatically converted into the values of PV, PP, MV, and MP. Previous studies have shown high reliability and validity of this linear position transducer [intra-class correlation coefficient (ICC) 0.970 to 0.988] for all variables measured in this study, with PP showing the highest coefficient of variation (CV; 13%; Garnacho-Castaño et al., 2015). All subjects completed the described testing protocol that was carefully replicated in subsequent experimental sessions.

#### Statistical Analysis

All statistical analysis were performed using Statistica 9.1. Results are presented as means with standard deviations. The Shapiro-Wilk’s, Levene’s, and Mauchly’s tests were used in order to verify the normality, homogeneity, and sphericity of the sample data variances, respectively. Differences between the E/E/E, S/E/E, and S/S/E conditions were examined using repeated measures two-way ANOVA (3 conditions x 3 repetitions). The statistical significance was set at *p* < 0.05. The effect size was determined by partial eta squared (*η*^2^). Partial eta squared values were classified as small (0.01–0.059), moderate (0.06–0.137), and large (>0.137). In the event of statistically significant main effect, the Tukey’s test were conducted to locate the differences between mean values. For pairwise comparisons, the effect sizes (Hedges’ g) were reported where appropriate. Parametric effect sizes were defined as large (g > 0.8); moderate (g between 0.8 and 0.5); small (g between 0.49 and 0.20); and trivial (g < 0.2). Percent changes with 95% confidence intervals (95CI) were also calculated. Statistical significance was set at *p* < 0.05.

## Results

The two-way repeated measures ANOVA showed a statistically significant interaction effect for PP (*p* = 0.03; *η*^2^ = 0.26) and for PV (*p* = 0.04; *η*^2^ = 0.24). The two-way repeated measures ANOVA showed a statistically significant main effect of condition for PP (*p* = 0.04; *η*^2^ = 0.30) and for PV (*p* = 0.02; *η*^2^ = 0.35).

**Figure 1:**
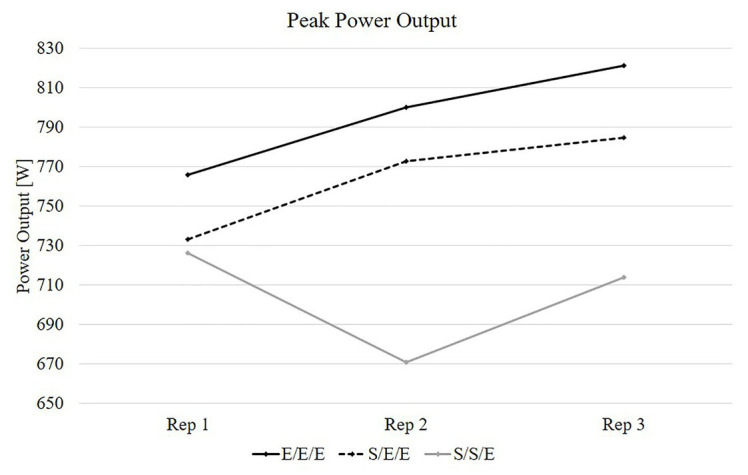
Result of peak power output for the three experimental movement tempos. E/E/E – 1st repetition explosive, 2nd repetition explosive, 3rd repetition explosive; S/E/E – 1st repetition slow, 2nd repetition explosive, 3rd repetition explosive; S/S/E – 1st repetition slow, 2nd repetition slow, 3rd repetition explosive.

**Figure 2:**
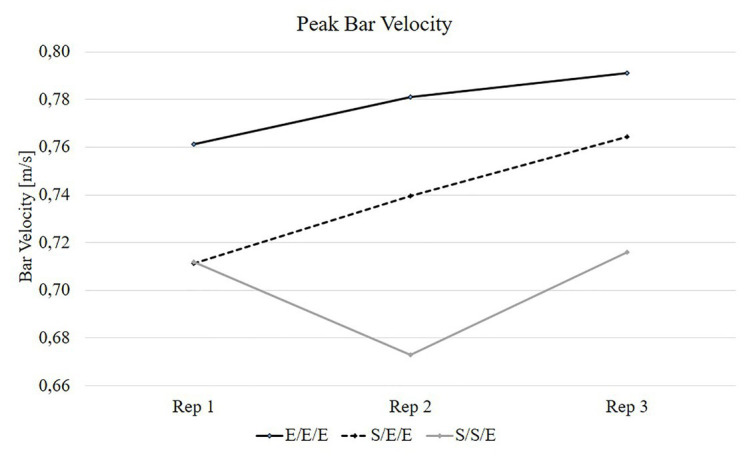
Results of peak bar velocity for the three experimental movement tempos. E/E/E – 1st repetition explosive, 2nd repetition explosive, 3rd repetition explosive; S/E/E – 1st repetition slow, 2nd repetition explosive, 3rd repetition explosive; S/S/E – 1st repetition slow, 2nd repetition slow, 3rd repetition explosive.

The *post hoc* analysis for interaction revealed that PP was significantly higher in the 2nd and 3rd repetition for the E/E/E compared with the S/S/E (*p* < 0.01 for both) and significantly higher in the 2nd repetition for the S/E/E compared with the S/S/E (*p* < 0.01; Table 1; Figure 1). The *post hoc* analysis for interaction revealed that PV was significantly higher in the 2nd and 3rd repetition for the E/E/E compared with the S/S/E (*p* < 0.01 for both) and significantly higher in the 2nd repetition for the S/E/E compared with the S/S/E (*p* < 0.01; Table 2; Figure 2).

**Table 1:** A comparison of the three experimental movement tempos in power output variables.

Condition	Peak power output			Mean power output		
	1st	2nd	3rd	1st	2nd	3rd
E/E/E	766 ± 165 (648 to 884)	800 ± 185 (668 to 933)^*^	821 ± 184 (690 to 953)^*^	507 ± 86 (446 to 569)	541 ± 102 (468 to 614)	542 ± 83 (482 to 602)
S/E/E	733 ± 150 (626 to 841)	773 ± 160 (659 to 887)^†^	785 ± 129 (692 to 877)	522 ± 87 (460 to 584)	559 ± 104 (484 to 633)	558 ± 103 (484 to 632)
S/S/E	726 ± 164 (609 to 843)	671 ± 123 (583 to 759)^*^^,^^†^	714 ± 187 (580 to 847)^*^	498 ± 74 (446 to 551)	493 ± 70 (443to 543)	520 ± 108 (443 to 597)
Effect size												
E/E/E vs. S/E/E	0.20	0.16	0.23	0.17	0.17	0.17
E/E/E vs. S/S/E	0.24	0.82	0.58	0.11	0.55	0.23
S/E/E vs. S/S/E	0.05	0.71	0.44	0.30	0.74	0.36

Results are mean ± SD (95% confidence intervals). E/E/E – 1st repetition explosive, 2nd repetition explosive, 3rd repetition explosive; S/E/E – 1st repetition slow, 2nd repetition explosive, 3rd repetition explosive; S/S/E – 1st repetition slow, 2nd repetition slow, 3rd repetition explosive.

* Statistically significant differences *p* < 0.05.

† Statistically significant differences *p* < 0.05.

**Table 2:** A comparison of the three experimental movement tempos in bar velocity variables.

Condition	Peak bar output			Mean bar output		
	1st	2nd	3rd	1st	2nd	3rd
E/E/E	0.76 ± 0.12 (0.67 to 0.85)	0.78 ± 0.13 (0.69 to 0.87)^*^	0.79 ± 0.14 (0.69 to 0.89)^*^	0.55 ± 0.08 (0.49 to 0.60)	0.55 ± 0.08 (0.49 to 0.60)	0.58 ± 0.08 (0.53 to 0.64)
S/E/E	0.71 ± 0.10 (0.64 to 0.79)	0.74 ± 0.10 (0.67 to 0.81)^†^	0.76 ± 0.10 (0.70 to 0.83)	0.56 ± 0.09 (0.50 to 0.63)	0.60 ± 0.10 (0.53 to 0.67)	0.60 ± 0.10 (0.53 to 0.67)
S/S/E	0.60 ± 0.10 (0.53 to 0.67)	0.67 ± 0.12 (0.58 to 0.76)^*^^,^^†^	0.72 ± 0.16 (0.60 to 0.83)^*^	0.54 ± 0.09 (0.48 to 0.61)	0.54 ± 0.08 (0.48 to 0.59)	0.56 ± 0.11 (0.48 to 0.64)
Effect size												
E/E/E vs. S/E/E	0.45	0.34	0.25	0.12	0.22	0.23
E/E/E vs. S/S/E	0.35	0.88	0.47	0.12	0.50	0.21
S/E/E vs. S/S/E	0.00	0.63	0.30	0.22	0.66	0.40

Results are mean ± SD (95% confidence intervals). E/E/E – 1st repetition explosive, 2nd repetition explosive, 3rd repetition explosive; S/E/E – 1st repetition slow, 2nd repetition explosive, 3rd repetition explosive; S/S/E – 1st repetition slow, 2nd repetition slow, 3rd repetition explosive.

* Statistically significant differences *p* < 0.05.

† Statistically significant differences *p* < 0.05.

The *post hoc* analysis for main effect of condition revealed that PP and PV was significantly higher for the E/E/E compared with the S/S/E (*p* = 0.04; *p* = 0.02; respectively).

There was no significant interaction effect for MP (*p* = 0.20; *η*^2^ = 0.15) and for MV (*p* = 0.27; *η*^2^ = 0.13).

## Discussion

The main finding of this study was that the different distribution of movement tempo during a set has a significant impact on power output and bar velocity in the bench press exercise at 60%1RM. The results of our study indicate that PP and PV generated during the set was significantly higher for the E/E/E compared to the S/S/E condition. The significantly higher PP and PV was observed in the 2nd and 3rd repetition of a set in which the E/E/E tempo was used compared to the S/S/E tempo of movement. Furthermore, we also observed significantly higher PP and PV in the 2nd repetition of a set in which the S/E/E tempo was used compared to the S/S/E. However, there was no significant change in PP and PV between E/E/E and S/E/E tempos. Therefore, the use of one slow repetition at the beginning of a set does not decrease the level of power output in the 3rd repetition. Furthermore, the results of our study did not show significant differences between particular tempos of movement for MP and MV.

To the best of our knowledge, there are no available data regarding acute power output and bar velocity changes during a resistance exercise with contrast movement tempo, which limits the possibility of comparing our results with other studies. Nevertheless, significant knowledge and training clues can be derived from the current data. Greater values of PP and PV for the E/E/E condition compared to the S/S/E condition indicates that different distribution of movement tempo during a set may be a factor affecting the level of power output. Despite that the last 3rd repetition was performed with maximal movement tempo during all three trials, there was a significant decrease in PP and PV for the S/S/E condition when compared to the E/E/E protocol. The significant decrease in PP and PV for the 3rd repetition following the use of the S/S/E tempo, compared to the E/E/E tempo can be attributed to the volume of effort. Changing the movement tempo has a significant effect on the TUT for each set and the entire training session (Wilk et al., 2020d). During the bench press exercise performed with the S/S/E tempo, the TUT for a set was significantly longer when compared to the S/E/E tempo, and to the E/E/E movement tempo (~22 s; ~14 s; ~6 s; respectively). According to McBride et al. (2009) and Wilk et al. (2018a, 2020d), TUT is one of the indicators of resistance training volume. Longer TUT increases metabolic stress and endocrine post-exercise responses (Wilk et al., 2018b, 2020d) and, therefore, potentially increases fatigue, which negatively affects power performance in subsequent repetitions, that was observed in the 3rd repetition for the S/S/E tempo when compared to the E/E/E movement tempo. The decrease in power output and bar velocity for the longer TUT recorded in our study is partially consistent with previous results published by Wilk et al. (2019b) and Wilk et al. (2020c). Wilk et al. (2019b) showed a significant decrease in the power output and bar velocity for slow eccentric contractions and longer TUT (6/0/X/0) when compared to fast eccentric contractions and shorter TUT (2/0/X/0). A further decrease in PP and PV for the S/S/E condition compared to the E/E/E can be related not only to increased fatigue but may also be related to the reduction of potentiating effect of the previous repetition. The study by the Wilk et al. (2020c) showed that the post-activation performance enhancement in successive sets was less pronounced following slower tempo of movement (6/0/X/0 vs. 2/0/X/0). Therefore, it can be suggested that a similar effect could occur between successive repetitions, where longer two previous repetitions decrease post-activation performance enhancement in the 3rd successive repetition. The result of this study also indicates a significant decrease in PP and PV during the 2nd repetition for the S/S/E tempo when compared to the E/E/E and to the S/E/E condition. However, this decrease in PP and PV was related to the intentional slowdown of the concentric movement, resulting from the applied research procedure.

Despite greater TUT observed in the S/E/E tempo compared to the E/E/E tempo, the results did not show any negative effects on power output and bar velocity. This may be caused by the lower TUT differences between tempos (E/E/E ~6 s; S/E/E ~14 s) and the fact that such brief efforts, which were performed during the S/E/E tempo, are fueled mainly by phosphocreatine (Bird et al., 2005). Therefore, the use of one slow repetition at the beginning of a set does not limit the power output capacity during subsequent repetitions. It should be noted that there was no statistical differences in the 3rd repetition between the S/E/E and the S/S/E conditions. Furthermore, the ES between S/E/E and S/S/E conditions was similar to those observed between the E/E/E and the S/E/E conditions (0.29–0.44 vs. 0.17–0.30; respectively); this indicates that the difference in movement tempo of one repetition does not decrease the power output performance in subsequent repetitions. However, when the difference in movement tempo concerns two repetitions, significant changes in PP and PV between E/E/E and S/S/E occur, which indicates that there is a continuum of effects being produced. Further, as suggested by Wilk et al. (2019b, 2020c), when using a controlled tempo of movement in more than one set, the power output in the concentric contraction depends not only on TUT but also on the ratio of the TUT in a particular set to the duration of the rest interval. In case of contrast movement tempo, it seems that the availability of phosphocreatine, and therefore the achieved TUT, is the main factor, which promotes the maintenance of power output in subsequent repetitions. Therefore, the use two slow repetitions, but with, e.g., 3/0/3/0 tempo (not as in the presented study with a 5/0/5/0 tempo), could allow the maintenance of power in the last 3rd repetition.

According to the American College of Sports Medicine (2009), untrained individuals should use slow and moderate movement tempos (Hay et al., 1983; Lachance and Hortobagyi, 1994; Keeler et al., 2001; Munn et al., 2005; Neils et al., 2005). For intermediate sports level, it is recommended that moderate tempos should be used (Hay et al., 1983; Lachance and Hortobagyi, 1994; Keeler et al., 2001; Munn et al., 2005; Neils et al., 2005), and for advanced athletes, a variety of tempos from slow to fast are recommended. However, different movement tempos cause different physiological responses. Slower movement tempo increases maximal exercise duration (Wilk et al., 2019a) but, at the same time, decreases the number of possible repetitions performed and limits the frequency and efficiency of the stretch-shortening cycle (Wilk et al., 2019b), which can be counterproductive in sports requiring explosive movements. Further, slow movement tempo increases post-exercise hormonal and metabolic responses (Goto et al., 2008; Wilk et al., 2018b). In contrast, fast movement tempo increases the amount of performed repetitions (Sakamoto and Sinclair, 2006; Wilk et al., 2018a), the level of generated power (Wilk et al., 2019b), as well as an increase of EMG amplitude (Sakamoto and Sinclair, 2012; Sampson et al., 2014). Therefore, the use of contrasting movement tempo in one set can combine the benefits of fast and slow movement tempo. However, considering that the TUT gradually increases with every repetition (especially when slower movement tempo is used), a higher number of slower repetitions at the beginning of a set may cause an additional increase of fatigue and a decrease in power output in subsequent explosive repetitions from what was observed in the S/S/E condition. In this case, the coach and athlete can consider using a slower movement tempo only during the first repetition (which will cause an increase in muscle activation and lengthening of TUT) with an explosive final repetition (optimal for development of power). Such a contrast tempo can be an effective alternative compared with traditional resistance training, which could help athletes break through plateaus and prevent training monotony (Krzysztofik et al., 2019; Wilk et al., 2020e). Moreover, due to the longer TUT during S/E/E or S/S/E tempos, longer rest intervals may be required between sets compared to the E/E/E tempo, when the training objective is power development.

Although the results of the present study showed that different movement tempo distribution during a set of resistance exercise may be used to enhance performance, there are certain limitations, which should be addressed. Although the results showed that PP and PV in the 3rd repetition was significantly lower for the S/S/E tempo when compared to the E/E/E tempo, the causes of these changes could not be determined and explained due to the lack of physiological and biomechanical evaluations, which could provide possible explanations. Moreover, the results of this study only apply to the bench press exercise performed at 60%1RM and may not translate into a different tempo distribution, repetition duration, exercise type, or other loads and grip widths used, which requires further research. It should be remembered that in order to optimize the development of power, it is recommended not only to use a fast or explosive movement tempo but also to use a combination of different external loads and grip widths that cause changes in the range of joint motion (Williams et al., 2018). Therefore, further research is required in this area.

## Practical Implications

The present study showed that different distribution of movement tempo during a set of resistance exercise has a significant impact on power performance of the upper limbs. However, the observed decrease in power output and bar velocity in the 3rd repetition applies only to peak values and only to the S/S/E tempo. Such changes in the 3rd repetition were not observed with the S/E/E tempo. Furthermore, significant changes in MP and MV between all three tested tempos were not observed. Therefore, the use of slow repetitions at the beginning of a set, with longer TUT, can be effective in stimulating muscle strength and hypertrophy. However, the possibility of using a slow movement tempo, especially in the concentric contraction, is limited by the external load. Therefore, the use of slow movement tempo forces to a decrease of external load, which may reduce strength gains following a long-term resistance training. Previous studies indicate that heavier loads produce greater strength gains than lighter loads, although the velocity of movement could be faster (Schoenfeld et al., 2015b; Gonzalez, 2016; Lasevicius et al., 2019). In this case, athletes may consider using slow movement tempo in the first repetition only in the eccentric contraction, using fast or explosive tempo in the concentric phase of movement (e.g., 6/0/X/0), and using subsequent repetitions with maximal tempo in both phases (X/0/X/0). During the eccentric contraction, even at heavier loads, the movement tempo can be controlled to some extent. For power development, the training with the intention of moving the load explosively is believed to be optimal for power adaptations, irrespective of the contraction type, load, or actual movement velocity of the exercises used (Behm and Sale, 1993; Fielding et al., 2002; Cormie et al., 2011; Haff and Stone, 2015; Fernandes et al., 2018). Therefore, the use of slow repetition will reduce the velocity of movement, which will have a significant negative effect on acute power output, and, as a consequence, can potentially limit the possibilities of power development. However, it has not been investigated whether the use of one or two slow repetitions at the beginning of a multi-repetition set decreases long-term power development, which requires further studies.

The use of different movement tempo distribution during a set can be useful, especially during complex resistance training (Wilk et al., 2020d). The slow movement tempo in the first repetition (which will cause an increase in muscle activation and lengthening the TUT) and an explosive movement in the next repetitions (which will increase concentric velocity) should be optimal for hypertrophy development and power output simultaneously. It is also possible to use an inverse tempo distribution to those presented in this study, where fast repetitions will be performed at the beginning of the set and slow repetitions at the end of the set. Such a complex resistance training may be an effective alternative compared with traditional resistance protocols, especially when the time to perform specific resistance training goals is limited. Further, it should be noted that, despite the lack of changes in MP and MV between the used tempos, the slower repetitions during the S/S/E or the S/E/E tempo by extending TUT could induce additional physiological responses (not evaluated in the present study), such as metabolic stress, and increased endocrine responses. Therefore, maintaining MP and MV while increasing physiological responses during resistance exercise with slower 1st, or 1st two, repetitions, can be a significant factor determining the level of post-exercise adaptive changes.

## Conclusion

The results of the present study indicate that two 1st slow repetitions significantly decreased PP and PV in the 3rd repetition of a bench press exercise at 60%1RM. The decrease in PP and PV in the 3rd repetition for the S/S/E tempo, compared to the E/E/E tempo, may be explained by the increased TUT for the set containing two slow repetitions. However, such a decrease in the 3rd repetition was not observed when only one slow repetition was used in a set. Therefore, the use of a slow repetition at the beginning of a set could increase the duration of a set without substantial negative consequences on power performance. These findings expand the scientific knowledge related to movement tempo during resistance exercise. The preliminary results indicate that different tempo distribution may have significant practical implications for coaches and athletes.

## Data Availability Statement

The raw data supporting the conclusions of this article will be made available by the authors, without undue reservation.

## Ethics Statement

The studies involving human participants were reviewed and approved by the Bioethics Committee for Scientific Research, at the Academy of Physical Education in Katowice, Poland (10/2018). The patients/participants provided their written informed consent to participate in this study.

## Author Contributions

MW, JJ, and MK: study conception and design. JJ, AF-S, AR-B, and MB: acquisition of data. MW, MK, AF-S, and PS: analysis and interpretation of data. MW, MK, and AF-S: drafting of manuscript. MW and AZ: critical revision. All authors contributed to the article and approved the submitted version.

### Conflict of Interest

The authors declare that the research was conducted in the absence of any commercial or financial relationships that could be construed as a potential conflict of interest.
