# Corrigendum: Contrast Tempo of Movement and Its Effect on Power Output and Bar Velocity During Resistance Exercise

**DOI:** 10.3389/fphys.2021.664495

**Published:** 2021-03-12

**Authors:** Michal Wilk, Jakub Jarosz, Michal Krzysztofik, Aleksandra Filip-Stachnik, Marcin Bialas, Agata Rzeszutko-Belzowska, Adam Zajac, Petr Stastny

**Affiliations:** ^1^Institute of Sport Sciences, Jerzy Kukuczka Academy of Physical Education in Katowice, Katowice, Poland; ^2^Faculty of Physical Education, Gdańsk University of Physical Education and Sport, Gdańsk, Poland; ^3^College of Medical Sciences, Institute of Physical Culture Studies, University of Rzeszów, Rzeszów, Poland; ^4^Department of Sport Games, Faculty of Physical Education and Sport, Charles University, Prague, Czechia

**Keywords:** resistance exercise, cadence, time under tension, velocity of movement, duration of repetition

In the original article as published in the “Materials and Methods” and the “Experimental Sessions” section, the slow movement tempo description was incorrectly written as “5/0/5/0”. The correct description should be written as “5/0/X/0”. The correct paragraphs are below:

“The researchers examined the impact of different movement tempo distribution during a single set of the bench press exercise on power output and bar velocity. The experiment was performed following a randomized crossover design, where each subject performed three different testing protocols in random and counterbalanced order, one week apart: with an explosive movement tempo in each of three repetitions (E/E/E = explosive, explosive, explosive); with slow tempo of movement in the first repetition and explosive one in the next two repetitions (S/E/E = slow, explosive, explosive); and with a slow tempo of movement in the first two repetitions and explosive tempo in the last repetition of the set (S/S/E = slow, slow, explosive). The slow repetitions were performed with a 5/0/X/0 movement tempo, while the explosive repetitions were performed with an X/0/X/0 movement tempo. During each experimental session, the participants performed one set of three repetitions at 60%1RM. Before the main tests, one familiarization session was allowed. One week before the first main session, maximal bench press strength (1 repetition maximum-1RM) was evaluated. The following variables were measured only in the third repetition using a linear position transducer: peak power output (PP), mean power output (MP), peak bar velocity (PV) and mean bar velocity (MV). All testing sessions were performed in the Strength and Power Laboratory at the Academy of Physical Education in Katowice, Poland.”

“During the explosive repetition a X/0/X/0 movement tempo was used, while during the slow repetition protocol, the 5/0/X/0 movement tempo was used. During each testing protocol the subject performed one set of the bench press at 60%1RM, following a metronome guided movement tempo (Korg MA-30, Korg, Melville, New York, USA). The external load and number of performed repetitions was determined in accordance with the recommendation of ACSM (2009) concerning power training. A linear position transducer system (Tendo Power Analyzer, Tendo Sport Machines, Trencin, Slovakia) was used for the evaluation of bar velocity (Garnacho-Castaño et al., 2015). Measurements were made for only the third repetition and automatically converted into values of peak bar velocity (PV), peak power output (PP) mean bar velocity (MV) and mean power output (MP). Previous studies have shown high reliability and validity of this linear position transducer (intra-class correlation coefficient [ICC] 5 0.970 to 0.988) for all variables measured in this study, with PP showing the highest coefficient of variation (CV) (13%) (Garnacho-Castaño et al., 2015). All subjects completed the described testing protocol that was carefully replicated in subsequent experimental sessions.”

In the “Discussion” section, the movement tempo was incorrectly written as (~22s; ~14s; ~6s; respectively). The correct movement tempo should be written as “(~14s; ~10s; ~6s; respectively)”. The correct paragraph is below.

“To the best of our knowledge, there are no available data regarding acute power output and bar velocity changes during a resistance exercise with contrast movement tempo, which limits the possibility of comparing our results with other studies. Nevertheless, significant knowledge and training clues can be derived from the current data. Greater values of PP and PV in the last repetition for E/E/E condition compared to the S/S/E condition indicates that different distribution of movement tempo during a set may be a factor affecting the level of power output. The significant decrease in PP and PV following the use of the S/S/E tempo, compared to the E/E/E tempo can be attributed to the volume of effort. Changing the movement tempo has a significant effect on the TUT for each set and the entire training session (Wilk et al., 2020d). During the bench press exercise performed with the S/S/E tempo the TUT for a set was significantly longer, when compared to the S/E/E tempo and to the E/E/E movement tempo (~14s; ~10s; ~6s; respectively). According to McBride et al. (2009); Wilk et al. (2018a, 2020d), TUT is one of the indicators of resistance training volume. Longer TUT increases metabolic stress and endocrine post-exercise responses (Wilk et al., 2018b, 2020d) and therefore, potentially increases fatigue, which negatively affects power performance in subsequent repetitions, what was observed for the S/S/E tempo when compared to the E/E/E movement tempo. The decrease in power output and bar velocity for the longer TUT recorded in our study is partially consistent with previous results published by Wilk et al. (2019b) and Wilk et al. (2020c). Wilk et al. (2019b) showed a significant decrease in the power output and bar velocity for slow eccentric contractions and longer TUT (6/0/X/0) when compared to fast eccentric contractions and shorter TUT (2/0/X/0). A further decrease in PP and PV for S/S/E condition compared to the E/E/E can be related not only to increased fatigue, but may also be related to the reduction of potentiating effect of the previous repetition. The study by the Wilk et al. (2020c) showed that the post-activation performance enhancement in successive sets was less pronounced following slower tempo of movement (6/0/X/0 vs 2/0/X/0). Therefore it can be suggested that a similar effect could occur between successive repetitions, where longer two previous repetitions decrease post-activation performance enhancement in the 3rd successive repetition.”

The authors apologize for this error and state that this does not change the scientific conclusions of the article in any way. The original article has been updated.

